# Editorial: Dimensions of Health Information Quality

**DOI:** 10.3389/fpubh.2020.00223

**Published:** 2020-06-16

**Authors:** Pietro Ghezzi, Elizabeth Ford

**Affiliations:** Brighton and Sussex Medical School, Brighton, United Kingdom

**Keywords:** health records, patient data, websites, social media, literacy

Where is the life we have lost in living? Where is the wisdom we have lost in knowledge? Where is the knowledge we have lost in information? Eliot (1)

The data-information-knowledge-wisdom hierarchy, which we find *in nuce* in Eliot's poem, is a well-known theoretical model where with data and information we build knowledge about a subject, and then being able to apply this knowledge is called wisdom (2). One key issue in making use of information is its quality, and this Research Topic covers various aspects of health information quality.

A context in which the term information is used in medicine relates to biomedical data used for research purposes, and this can be in the form of electronic health records, self-tracked data (e.g., with mobile apps), and online discussion sites. Ford et al. explore an important aspect of the use of “big data” represented by electronic health records, indicating ways to address the issue of completeness of the information recorded. Their paper analyses the use of a Bayesian approach to address the issue of missingness in electronic health records. If information quality can be an issue in electronic health records produced by health professionals, this is an even greater problem in the case of patient-generated data. Several digital tools now allow tracking health-related activities, fitness, and wellbeing. West et al. present a systematic review of the literature on self-tracked data. The study discusses the importance of data quality in the context of their use in clinical practice to diagnose or manage conditions and conclude that, in the short term, their use in managing conditions is probably more realistic than their diagnostic use. Online discussion sites can also represent an important source of data health research and Smith et al., in their Opinion article, describe the promises and limitations of this approach.

Two other articles analyse the uses and consequences of digital health information. Reder et al. address the problem of decision aids to help people making health-related decision, particularly about screening options. Often, screening tests, such as mammography for breast cancer, present a complex mix of potential benefits and possible risks when offered to healthy people. It is therefore important to offer high quality information to individuals considering taking up an offer of screening. Different ways of communicating high quality information have been trialed (pamphlets, videos), but in this study the authors devised a web-based decision aid for women invited to a mammography screening, and they investigate whether this is equally useful to women with different degrees of eHealth literacy. Aslam et al. ask why many people believe that antioxidant supplements can improve or prevent disease conditions and which ones. Using search engines to collect a significant sample of webpages mentioning antioxidants, they find that antioxidants are often described as useful for broad indications and the information available is largely presented from commercial sources or news outlets. This raises the issue of information quality not only in terms of intrinsic criteria such as completeness, accuracy, and reliability, but also in terms of the ethic responsibility of information providers and gatekeepers to avoid commercially-biased mis-/dis-information.

Taken together this set of studies/papers showcases the range of health information on which clinicians, researchers, and the public may base their decisions about health and healthcare. It exposes numerous issues with information quality which may materially affect the decisions that people make. It is important, in this digital age, that population health literacy, particularly understanding the quality of health information, keeps up with the shifting landscape of health information sources used by patients and the public.

## Author Contributions

All authors listed have made a substantial, direct and intellectual contribution to the work, and approved it for publication.

## Conflict of Interest

The authors declare that the research was conducted in the absence of any commercial or financial relationships that could be construed as a potential conflict of interest.
